# Supplementary material for: Partnership preferences, economic drivers, and health consequences of Gambian men’s interactions with foreign tourists: A mixed methods study
Source: PLOS Glob Public Health. 2023 Feb 28;3(2):e0001115. doi: 10.1371/journal.pgph.0001115 (PMC10021562; doi:10.1371/journal.pgph.0001115)
Supplement: S1 Text — (DOCX) [file pgph.0001115.s001.docx]

# **S1 Text: Quantitative question scales**

**Household hunger scale**

- In the past 4 weeks, did it happen that there was no food to eat of any kind in your house because of lack of resources to get food?
- In the past 4 weeks, did it happen that you or any household member went to sleep at night hungry because there was not enough food?
- In the past 4 weeks, did it happen that you or any household member went a whole day or night without eating anything at all because there was not enough food?

Responses*: Never, rarely, sometimes, often*

**WHO wellbeing scale**

- I am going to ask you some questions about how you have felt in the last two weeks. Please indicate for each of the five statements which is closest to how you have been feeling over the last two weeks.
- Over the last two weeks… Did you feel cheerful and in good spirits?
- Over the last two weeks… Did you feel calm and relaxed?
- Over the last two weeks… Did you feel active and strong?
- Over the last two weeks… Did you wake up feeling fresh and rested?
- Over the last two weeks… Your daily life has been full of things that interest you?

Responses: *At no time, Some of the time, Less than half of the time, More than half of the time, Most of the time, All of the time*

**HIV knowledge tool**

Here are some statements about protection against HIV and STIs. For each statement please tell me whether you think it is true or false:

- Vaginal fluids can protect women from HIV.
- Coughing and sneezing openly DOES NOT spread HIV.
- A person can get HIV by sharing a glass of water with someone who has HIV.
- A woman can get HIV or STIs if she has anal sex with a man
- Showering, or washing one’s genitals/private parts, after sex keeps a person from getting HIV or STIs.
- It is possible for a HIV positive mother to give birth to a HIV negative child
- A person can get HIV or STIs by sitting in a hot tub or a swimming pool with a person who has HIV or STIs.
- Being circumcised reduces a man's chance of getting HIV from a woman
- If a HIV positive person takes HIV treatment regularly, they are less likely to transmit HIV to a sexual partner

Responses: *True, false, don’t know*

**STI syndromic tool**

Now I am going to ask you about the sexual health needs you might have, and any symptoms you may have had. First, during the last 12 months, have you had a disease which you got through sexual contact?

Sometimes men have a sore or ulcer near their penis. During the last 12 months, have you had a sore or ulcer on or near your penis?

Responses: *Yes, No*
