# Supplementary material for: Partnership preferences, economic drivers, and health consequences of Gambian men’s interactions with foreign tourists: A mixed methods study
Source: PLOS Glob Public Health. 2023 Feb 28;3(2):e0001115. doi: 10.1371/journal.pgph.0001115 (PMC10021562; doi:10.1371/journal.pgph.0001115)
Supplement: S2 Text — (DOCX) [file pgph.0001115.s002.docx]

# **S2 Text: Qualitative topic guide for IDI and FGDs**

**Interview guide for Gambian men**

**ISSUES: The impact of tourism on sexual networks, health, and health system utilisation in The Gambia and the UK**

This topic guide is shown here as a sample for the in-depth interviews and focus groups. It may be modified following the results from previous interviews or focus groups. The interviews will not necessarily cover all the topics or follow the order of the topic guide, as it will be adapted to each participant.

**I. Background information**

Participant ID:

Location:

Date and time:

**II. Ice-breaker questions**

Can you tell me what you do most days?

How long have you been doing it?

What are your views on the health system in your country and district?

**III. Perspectives of bumsters**

- What are your experiences of seeking health care here?
  - What sorts of things have you sought care for? (Probe for specific diseases or health issues)
  - Do you think you get the care that you need?
  - Have you had any particularly good or bad experiences seeking care here?
- Can you describe to me what being a bumster is?
  - What leads to people becoming bumsters?
  - What do bumsters do day-to-day?
  - What makes people stop being bumsters?
- How do bumsters make friends with tourists?
  - What is the purpose of making friends with tourists?
  - If a bumster makes good friends with a tourist, what are his expectations after the tourist returns home? (probe for source of information of “successful” bumsters)
- Do bumsters have sex with tourists often?
  - How are sexual relationships with tourists different than with Gambian partners?

(Probe for condom use, type and frequency of activity)

What happens outside of tourist season?

- - Do you think bumsters are at risk of sexually transmitted infections or HIV?
- What do you think the government of The Gambia should do about the practices of bumsters?
  - Should it be encouraged or discouraged? How?
- Are there any other issues that you would like to discuss?

**Thank you**
