# Supplementary material for: Partnership preferences, economic drivers, and health consequences of Gambian men’s interactions with foreign tourists: A mixed methods study
Source: PLOS Glob Public Health. 2023 Feb 28;3(2):e0001115. doi: 10.1371/journal.pgph.0001115 (PMC10021562; doi:10.1371/journal.pgph.0001115)
Supplement: S1 Table — (DOCX) [file pgph.0001115.s004.docx]

# **S1 Table: Additional results**

|  | **Mean** | **SD** | **Median** | **N** | **%** | **Obs.** |
| --- | --- | --- | --- | --- | --- | --- |
| Age | 39.2 | 9.58 | 38 |  |  | 242 |
| Secondary education as highest education level | 0.7 | 0.46 |  | 169 | 70% | 242 |
| Would find it more than a little difficult to pay 350GMD bill | 0.85 | 0.35 |  | 206 | 85% | 242 |
| Occupation |  |  |  |  |  |  |
| *Tour guide* |  |  |  | 48 | 20% | 240 |
| *Tourist taxi driver* |  |  |  | 48 | 20% | 240 |
| *Bumster / chanter (chancer)* |  |  |  | 20 | 8% | 240 |
| *Fruit seller* |  |  |  | 3 | 1% | 240 |
| *Horse rider* |  |  |  | 5 | 2% | 240 |
| *Juice presser* |  |  |  | 27 | 11% | 240 |
| *No occupation / not registered* |  |  |  | 9 | 4% | 240 |
| *Other* |  |  |  | 69 | 29% | 240 |
| *Restaurant / bar worker* |  |  |  | 10 | 4% | 240 |
| *Brothel owner* |  |  |  | 1 | 0% | 240 |
| Monthly income (GMD) | 3468 | 3960 | 3000 |  |  | 242 |
| Time working with tourists (months) | 110 | 97 | 90 |  |  | 242 |
| Months per year working with tourists | 7.4 | 2.67 | 6 |  |  | 242 |
| Moderate or high household hunger |  |  |  | 35 | 15% | 240 |
| **Sexual history** |  |  |  |  |  |  |
| Currently cohabiting |  |  |  | 123 | 51% | 242 |
| Age at first sex | 30.4 | 45 | 22 |  |  | 242 |
| Number of lifetime sexual partners | 5.4 | 6.37 | 4 |  |  | 239 |
| Ever had sex with tourist |  |  |  | 122 | 50% | 242 |
| Condom use (last sex) |  |  |  | 95 | 39% | 242 |
| Condom use (always) |  |  |  | 74 | 31% | 242 |
| Source of last condom |  |  |  |  |  |  |
| *Pharmacy* |  |  |  | 49 | 52% | 95 |
| *Other shop* |  |  |  | 2 | 2% | 95 |
| *Health clinic (public)* |  |  |  | 3 | 3% | 95 |
| *Health clinic (private)* |  |  |  | 2 | 2% | 95 |
| *Friend/relative* |  |  |  | 25 | 26% | 95 |
| *My partner gave it to me* |  |  |  | 11 | 12% | 95 |
| *Other (Specify)* |  |  |  | 2 | 2% | 95 |
| Age difference of last sexual partner | 7.8 | 9.8 | 8 |  |  | 241 |
| Origin of last sexual partner |  |  |  |  |  |  |
| *Live here - of Gambian origin* |  |  |  | 163 | 67% | 242 |
| *Live here - of other origin* |  |  |  | 27 | 11% | 242 |
| *Visitor - but stay in the Gambia for long periods of time* |  |  |  | 2 | 1% | 242 |
| *Visitor - came for a short period of time* |  |  |  | 45 | 19% | 242 |
| *Don't know/other* |  |  |  | 5 | 2% | 242 |
| Number of tourists had sex with | 2.2 | 3.06 | 1 |  |  | 122 |
| Condom use (last sex with tourist) |  |  |  | 75 | 61% | 122 |
| Condom use (always with tourists) |  |  |  | 64 | 52% | 122 |
| Number of acts with last tourist | 4.9 | 6 | 3 |  |  | 122 |
| Received gifts or money in return for sex |  |  |  | 43 | 35% | 122 |
| Relationship maintained after partner left |  |  |  | 73 | 60% | 122 |
| **Health and health seeking** |  |  |  |  |  |  |
| STI or symptoms in last 12 months |  |  |  | 22 | 9% | 242 |
| Ever taken HIV test |  |  |  | 91 | 38% | 242 |
| >1 alcoholic drink in last 30 days |  |  |  | 42 | 17% | 242 |
| Drank heavily at last sex |  |  |  | 12 | 5% | 242 |
| Drug use in last 3 months |  |  |  | 115 | 48% | 242 |
| Mental wellbeing (WHO-5) - total | 16.2 | 4.2 | 16 |  |  | 242 |
| Mental wellbeing - <50% aggregate score |  |  |  | 56 | 23% | 242 |
| Highly risk-loving (10/10) |  |  |  | 139 | 57% | 242 |
| HIV knowledge - v high (9/9) |  |  |  | 60 | 25% | 242 |
| HIV knowledge - low (<6/9) |  |  |  | 113 | 47% | 242 |
| Last care location |  |  |  |  |  |  |
| *Government hospital* |  |  |  | 82 | 34% | 242 |
| *Government health centre* |  |  |  | 53 | 22% | 242 |
| *Pharmacy* |  |  |  | 36 | 15% | 242 |
| *MRC Fajara* |  |  |  | 30 | 12% | 242 |
| Private hospital |  |  |  | 17 | 7% | 242 |

**Table A: Full descriptive statistics of quantitative sample**

|  | (1) | (2) | (3)  MMNL | |
| --- | --- | --- | --- | --- |
| Level | MNL | MNL |  |  |
|  |  |  | Coefficient | SD |
| **Condom use** |  |  |  |  |
| Condom always used | 0.673*** | 0.658*** | 88.457+ |  |
|  | (0.0614) | (0.061) |  |  |
| Condom used half of the time | 0.0827 | 0.0514 | -24.493*** | 24.970 |
|  | (0.0647) | (0.0638) | (6.611) | (11.172) |
| Condom never used | -0.756*** | -0.710*** | -63.964*** | 35.819 |
|  | (0.0763) | (0.0749) | (15.225) | (13.466) |
| **Money** |  |  |  |  |
| No money (0 dalasi) | -0.399*** |  |  |  |
|  | (0.1003) |  |  |  |
| 500 dalasi | 0.139** |  |  |  |
|  | (0.0663) |  |  |  |
| 1000 dalasi | 0.000303 |  |  |  |
|  | (0.0895) |  |  |  |
| 2000 dalasi | 0.260*** |  |  |  |
|  | (0.0934) |  |  |  |
| Continuous, 100 dalasi |  | 0.0201*** | 0.98*** | 0.0046 |
|  |  | (0.007) | (0.314) | (0.634) |
| **When paid** |  |  |  |  |
| Money given at first sex | 0.149* | .0132* | 1.512+ |  |
|  | (0.0776) | (0.0677) |  |  |
| Money given at the end of your partner's trip | -0.117 | -0.0956 | -3.908 | 4.637 |
|  | (0.0919) | (0.0858) | (4.951) | (9.996) |
| Money promised one week after partner leaves | -0.0327 | -0.0364 | 2.396 | 1.359 |
|  | (0.0719) | (0.0712) | (3.926) | (10.886) |
| **Relationship length** |  |  |  |  |
| One night | -0.728 | -0.155* | 20.55+ |  |
|  | (0.0880) | (0.083) |  |  |
| 3-4 nights whilst partner is on holiday | -0.379*** | -0.255** | -21.321*** | 80.244 |
|  | (0.113) | (0.106) | (5.376) | (17.51) |
| Relationship lasts for their trip & partner will come back to see you in a few months | -0.0728 | -0.155* | -8.741 | 35.583 |
|  | (0.0880) | (0.0830) | (5.515) | (13.504) |
| Relationship lasts for their trip & partner will invite you to Europe in a few months | 0.172** | 0.195** | 9.512* | 3.743 |
|  | (0.0825) | (0.0803) | (5.380) | (11.033) |
| **Partner age** |  |  |  |  |
| 30-40 years | 0.212*** | 0.194*** | 34.568+ |  |
|  | (0.0619) | (0.0607) |  |  |
| 40-50 years | -0.0127 | 0.00878 | -6.687* | 6.801 |
|  | (0.0576) | (0.0562) | (3.459) | (8.808) |
| 50-60 years | -0.200*** | -0.203*** | -27.881*** | 52.78 |
|  | (0.0692) | (0.0663) | (7.320) | (14.960) |
| **Opt-out** | 0.522*** | 0.412*** | -11.624** | 70.443 |
|  | (0.0666) | (0.0560) | (5.373) | (18.137) |
|  |  |  |  |  |
| Number of choices made | 1,843 | 1,843 | 1,843  1829.1  0.10 | |
| Log likelihood | 1840.7 | 1846.9 |  |  |
| Pseudo R2 | 0.09 | 0.09 |  |  |

Standard errors in parentheses. *** p<0.01, ** p<0.05, * p<0.1. Coefficients effects coded. For MNL models, omitted category estimates obtained through changing the omitted category for each attribute. +For MMNL models, omitted category coefficients recovered through calculation.

**Table B: Discrete choice experiment results**
