# Supplementary material for: Partnership preferences, economic drivers, and health consequences of Gambian men’s interactions with foreign tourists: A mixed methods study
Source: PLOS Glob Public Health. 2023 Feb 28;3(2):e0001115. doi: 10.1371/journal.pgph.0001115 (PMC10021562; doi:10.1371/journal.pgph.0001115)
Supplement: Attachment — Submitted filename: Response to technical check failure.docx [file pgph.0001115.s005.docx]

**Response to technical check email Sept 9^th^ 2022:**

**PLOS GPH - Partnership preferences, economic drivers, and health consequences of Gambian men's interactions with foreign tourists: A mixed methods study**

**Dear Editorial team – apologies for the delay in responding, we were awaiting a response from our ethics committee regarding data availability.**

 We note your Data Availability statement: "We will make quantitative datasets and analysis files fully publically available through LSHTM data compass with DOI. Qualitative data (transcripts) contain potentially identifiable information - these will not be made publicly available."

We need additional information to ensure your statement aligns with our data-sharing policy. Please address the following:

Please make your data publicly available and provide any DOIs, links, or accession numbers needed to directly access your data. We hope to hear from you soon.

**The cross-sectional survey data has been set to request access due to concerns about participant identifiability. The data set contains indirect identifiers (place of operation, age, gender, time period in locaton, time period working in industry) that might allow others, particularly those working in locations close to respondents, to determine the identify of a participant (or mistakenly attribute it to someone else). If a participant’s identity was determined, their survey responses may have social, career or other implications for them or any colleagues mentioned.**

**In addition, participants were told that information collected about them would be kept strictly confidential, and personal information would only be shared with certain individuals. The LSHTM ethics committee has advised that this does not allow for the data to be made publicly available.**

**Data requests submitted through the LSHTM data repository (**[**https://doi.org/10.17037/DATA.00003105**](https://doi.org/10.17037/DATA.00003105)**) are sent to the project team and LSHTM Research Data Management Service for consideration. The LSHTM RDM Service acts as independent advisor for data access requests, directing them to the relevant ethics committee or other institutional body as appropriate. We encourage data access requests to be submitted through the LSHTM data repository, to ensure they’re distributed to all named contacts. However, applicants can email** [**researchdatamanagement@lshtm.ac.uk**](mailto:researchdatamanagement@lshtm.ac.uk) **with the DOI for the dataset being requested if preferred.**

2. The resolution of Figure 1 is very low and somewhat difficult to read. It is important that our Editors and Peer Reviewers are able to read all parts of a submission. Please replace these figures with higher resolution copies.

Please provide separate figure files in .tif or .eps format only and ensure that all files are under our size limit of 10MB.

For more information about how to convert your figure files please see our guidelines: https://journals.plos.org/globalpublichealth/s/figures

**Thank you – we have updated these figures with higher resolution .tif files**

3. Some material included in your submission may be copyrighted. According to PLOS’s copyright policy, authors who use figures or other material (e.g., graphics, clipart, maps) from another author or copyright holder must demonstrate or obtain permission to publish this material under the Creative Commons Attribution 4.0 International (CC BY 4.0) License used by PLOS journals. Please closely review the details of PLOS’s copyright requirements here: PLOS Licenses and Copyright. If you need to request permissions from a copyright holder, you may use PLOS's Copyright Content Permission form.

Please respond directly to this email or email the journal office and provide any known details concerning your material's license terms and permissions required for reuse, even if you have not yet obtained copyright permissions or are unsure of your material's copyright compatibility.

Potential Copyright Issues:

Figure 2: Please confirm whether you drew the images / clip-art within the figure panels by hand. If you did not draw the images, please provide (a) a link to the source of the images or icons and their license / terms of use; or (b) written permission from the copyright holder to publish the images or icons under our CC-BY 4.0 license. Alternatively, you may replace the images with open source alternatives. See these open source resources you may use to replace images / clip-art:
- https://commons.wikimedia.org
- https://openclipart.org/

**Thank you – we have removed the images for which copyright is not held**

Figure 1: please (a) provide a direct link to the base layer of the map (i.e., the country or region border shape) and ensure this is also included in the figure legend; and (b) provide a link to the terms of use / license information for the base layer image or shapefile. We cannot publish proprietary or copyrighted maps (e.g. Google Maps, Mapquest) and the terms of use for your map base layer must be compatible with our CC-BY 4.0 license.

Note: if you created the map in a software program like R or ArcGIS, please locate and indicate the source of the basemap shapefile onto which data has been plotted.

If your map was obtained from a copyrighted source please amend the figure so that the base map used is from an openly available source. Alternatively, please provide explicit written permission from the copyright holder granting you the right to publish the material under our CC-BY 4.0 license.

Please note that the following CC BY licenses are compatible with PLOS license: CC BY 4.0, CC BY 2.0 and CC BY 3.0, meanwhile such licenses as CC BY-ND 3.0 and others are not compatible due to additional restrictions.

If you are unsure whether you can use a map or not, please do reach out and we will be able to help you. The following websites are good examples of where you can source open access or public domain maps:
* U.S. Geological Survey (USGS) - All maps are in the public domain. (http://www.usgs.gov)
* PlaniGlobe - All maps are published under a Creative Commons license so please cite “PlaniGlobe, http://www.planiglobe.com, CC BY 2.0” in the image credit after the caption. (http://www.planiglobe.com/?lang=enl)
* Natural Earth - All maps are public domain. (http://www.naturalearthdata.com/about/terms-of-use/)

**Thank you – we have followed the open street map reporting and referencing guidelines in the revised figure under licence CC BY-SA 2.0.**
